# Types of Interpersonal Bias: Measurement and Their Association With Health Among LGBTQ Older Adults

**DOI:** 10.1093/geroni/igaa057.2614

**Published:** 2020-12-16

**Authors:** Anyah Prasad, Karen Fredriksen Goldsen, Hyun Kim, Hailey Jung

**Affiliations:** 1 University of Massachusetts Boston, Boston, Massachusetts, United States; 2 University of Washington, Seattle, Washington, United States

## Abstract

Increasing diversity and rapidly evolving sociopolitical context is changing the face of bias experiences in contemporary America. Accordingly, literature has evolved into four unique ways of describing bias, namely lifetime discrimination and victimization, everyday discrimination, and microaggressions. However, there is less conceptual clarity on the way these constructs are operationalized, measured, and their relationship to one another and health outcomes. In this paper, we discuss the measurement of these four constructs and report their prevalence among a national sample of LGBTQ older adults (N=2,450). The correlation between these constructs varied between 0.36 to 0.63, which shows that despite considerable overlap they are measuring distinct aspects of bias experiences. Each of the constructs had a significant association with depression, general health and quality of life. When the four constructs were entered into a regression model, everyday discrimination (due to sexual and gender identity and other factors) emerged as the strongest predictor.

